# Impacto dos Baixos Níveis de Testosterona e SHBG sobre o Risco de Insuficiência Cardíaca: Uma Revisão Sistemática e Metanálise

**DOI:** 10.36660/abc.20250244

**Published:** 2025-10-29

**Authors:** Thiago Artioli, Layane Bonfante Batista, Kleber Franchini, José Nunes de Alencar

**Affiliations:** 1 Instituto Dante Pazzanese de Cardiologia São Paulo SP Brasil Instituto Dante Pazzanese de Cardiologia, São Paulo, SP – Brasil

**Keywords:** Insuficiência Cardíaca, Testosterona, Biomarcadores, Fatores de Risco

## Abstract

**Fundamento:**

Estudos sugerem uma possível associação entre baixos níveis de testosterona e globulina ligadora de hormônios sexuais (SHBG) e desfechos cardiovasculares adversos. No entanto, essa relação ainda não está claramente definida.

**Objetivos:**

Esta revisão sistemática teve como objetivo avaliar o valor preditivo dos níveis basais de testosterona, di-hidrotestosterona (DHT) e SHBG para a incidência de insuficiência cardíaca (IC), oferecendo uma compreensão mais aprofundada da influência hormonal sobre o risco de IC.

**Métodos:**

Realizamos uma busca abrangente nas bases de dados MEDLINE, Scopus e Web of Science para identificar estudos de coorte e estudos caso-controle aninhados que mediram os níveis hormonais em adultos sem diagnóstico prévio de IC. O risco de viés foi avaliado utilizando a ferramenta ROBINS-E. Razões de risco (*hazard risks*, HRs) e razões de chances (*odds ratios*, ORs) agrupadas foram estimadas por meio de modelos bivariados de efeitos aleatórios. Um nível de significância estatística de 0,05 foi adotado para todas as análises.

**Resultados:**

Dos 1209 artigos analisados, 738 permaneceram após a remoção de duplicatas. Seis estudos, totalizando 233 474 participantes (11 663 mulheres), atenderam aos critérios de inclusão. A redução de um desvio padrão nos níveis de testosterona foi modestamente associada ao aumento do risco de IC em homens (HR: 1,10; IC 95%: 1,03–1,17), mas não em mulheres (HR: 1,05; IC 95%: 0,98–1,16). Comparações entre quartis ou quintis não revelaram associações significativas, e os níveis de SHBG não foram preditores relevantes do risco de IC. A análise bayesiana forneceu evidência fraca para essa associação (fator de Bayes = 0,99).

**Conclusões:**

Esta metanálise sugere que níveis baixos de testosterona estão modestamente associados ao aumento do risco de IC em homens, destacando um aspecto potencialmente importante, porém pouco explorado, da saúde cardiovascular. A heterogeneidade nos desenhos dos estudos e nas características das populações, juntamente com as associações fracas observadas, reforça a necessidade de investigações mais rigorosas. Ensaios clínicos randomizados bem estruturados são essenciais para confirmar esses achados e esclarecer os mecanismos biológicos subjacentes.

## Introdução

A insuficiência cardíaca (IC) continua sendo um importante problema de saúde global, afetando mais de 60 milhões de pessoas em todo o mundo.^1,2^ Apesar dos avanços no tratamento da IC com fração de ejeção reduzida (ICFER), os pacientes continuam enfrentando riscos significativos de progressão da doença e desfechos adversos, mesmo com a terapia médica orientada por diretrizes.^3,4^

Os mecanismos neuro-hormonais, especialmente a ativação do sistema nervoso simpático e do sistema renina-angiotensina, são centrais na progressão da IC e estão intimamente ligados à morbidade e mortalidade.^5^ As terapias com foco nesses sistemas melhoraram o manejo da IC. No entanto, há evidências crescentes de que a redução na regulação de diversos hormônios e sinais metabólicos também contribui para a progressão da doença.

Aproximadamente 25% dos homens com IC crônica apresentam deficiência de testosterona, além de níveis reduzidos do androgênio adrenal Desidroepiandrosterona (DHEA) e seu sulfato. Essas deficiências estão correlacionadas com a gravidade da IC e associadas a sintomas como redução da massa muscular, caquexia, depressão e fadiga.^6-9^

Estudos recentes investigaram a relação entre os níveis séricos de testosterona, di-hidrotestosterona (DHT) e globulina ligadora de hormônios sexuais (SHBG, do inglês *sex hormone-binding globulin*) com a IC, abrangendo tanto a ICFER quanto a IC com fração de ejeção preservada (ICFEP).^10-13^ No entanto, esses achados são frequentemente contraditórios e carecem de uma análise abrangente, deixando lacunas na compreensão dos impactos específicos das deficiências hormonais sobre a IC.^14-17^

Com o envelhecimento da população e o aumento da prevalência de hipogonadismo, esclarecer o papel da testosterona e de hormônios relacionados na IC torna-se cada vez mais relevante. Esta revisão sistemática e metanálise busca avaliar quantitativamente o valor preditivo de níveis séricos baixos de testosterona, DHT e/ou SHBG para o risco futuro de IC, oferecendo uma visão mais clara sobre seu potencial como biomarcadores da progressão da IC e orientando futuras direções de pesquisa.

## Métodos

Este protocolo de revisão sistemática está registrado no Registro Prospectivo Internacional de Revisões Sistemáticas (PROSPERO) (número de registro CRD42024550371). O estudo não recebeu financiamento externo, e os autores declaram não haver conflitos de interesse. Todos os dados utilizados nesta revisão estão disponíveis com os autores.

### Critérios de elegibilidade dos estudos

Foram incluídos estudos de coorte ou estudos caso-controle aninhados que mediram os níveis séricos de testosterona, DHT e/ou SHBG em uma população geral sem histórico de IC. Relatos de caso, revisões e metanálises foram excluídos. Ao final do período de acompanhamento, indivíduos com níveis séricos baixos de testosterona, DHT e/ou SHBG foram comparados àqueles com níveis normais quanto à incidência de IC, incluindo ICFER e ICFEP. O ano de publicação e a duração específica do acompanhamento não foram considerados critérios de exclusão. Estudos que não mediram ou não relataram os desfechos de interesse foram considerados inelegíveis.

### Participantes

A população do estudo consistiu em adultos com níveis séricos basais de testosterona, DHT e/ou SHBG, que foram acompanhados posteriormente para o desenvolvimento de IC recém-diagnosticada, incluindo ICFER ou ICFEP. Os hormônios foram medidos por meio de imunoensaio ou espectrometria de massa. Foram excluídos pacientes com diagnóstico prévio de IC ou Infarto do Miocárdio (IM), mulheres grávidas, pacientes com diagnóstico atual ou anterior de câncer (por exemplo, câncer de próstata) e aqueles em uso de terapia de reposição de testosterona.

### Fonte de informação e estratégia de busca

Realizamos uma busca abrangente nas bases de dados MEDLINE, Scopus e Web of Science. Não foram aplicadas restrições quanto ao ano de publicação ou idioma, garantindo um escopo inclusivo. Os termos de busca utilizados, incluindo palavras-chave e descritores MeSH, foram: testosterona, DHT, SHBG, IC, ICFER e ICFEP. Esta revisão segue as diretrizes do PRISMA (Preferred Reporting Items for Systematic Reviews and Meta-Analyses).^18^

### Seleção e coleta de dados

Títulos e resumos foram avaliados de forma independente por dois revisores (T.A. e L.B.B.) para identificar estudos elegíveis, sendo quaisquer discordâncias resolvidas por um terceiro revisor. A triagem dos textos completos também foi realizada de forma independente por dois pesquisadores (J.A. e A.S.). A extração de dados, facilitada pelo software HubMeta,^19^ incluiu características dos estudos como autoria, ano de publicação, país de origem e delineamento do estudo. As variáveis principais extraídas incluíram níveis séricos de testosterona, DHT e/ou SHBG; os métodos laboratoriais utilizados; tipo de estudo; desfechos avaliados; fatores de confusão ajustados; idade média da população estudada; tempo de seguimento; país do estudo e ano de publicação. Os estudos precisavam fornecer informações suficientes para estimar uma razão de risco (*hazard ratio* – HR) com Intervalo de Confiança de 95% (IC95%). Foram extraídas as estimativas de HR ajustadas para o maior número possível de variáveis de confusão. As buscas e análises foram realizadas de forma independente pelos investigadores. A tabulação dos dados foi feita utilizando o software Excel.^20^

### Risco de viés

O risco de viés foi avaliado utilizando a ferramenta *Risk Of Bias In Non-randomized Studies of Exposures* (ROBINS-E).^2 1^

### Análises de sensibilidade

Nas análises de sensibilidade pré-especificadas, foram examinados dados relacionados a diferentes métodos laboratoriais de dosagem dos níveis de testosterona, DHT e SHBG. Além disso, para avaliar a influência de cada estudo nas estimativas agrupadas, realizamos uma análise de influência *leave-one-out* (LOO), removendo sequencialmente um estudo por vez e recalculando a HR resumida. Essa abordagem foi utilizada para verificar a robustez dos achados frente à variabilidade nas medições laboratoriais.

### Síntese dos dados e medidas de efeito

Em nossa metanálise, utilizamos um modelo bivariado de efeitos aleatórios para combinar as estimativas de razão de chances (*odds ratio*) entre os estudos. Esse modelo foi escolhido por sua capacidade de considerar tanto a variabilidade dentro dos estudos quanto entre eles. As análises foram realizadas utilizando os softwares R e OnlineMeta.^2 2^ Gráficos de floresta (*forest plots*) foram gerados para representar visualmente a distribuição das razões de chances entre os estudos e suas estimativas agrupadas, oferecendo um resumo gráfico claro dos resultados da metanálise. Um nível de significância estatística de 0,05 foi adotado para todas as análises.

## Resultados

### Estudos elegíveis

Nossa busca inicial nas bases de dados resultou em 1209 artigos. Após a remoção de duplicatas, restaram 738 artigos. Após a triagem de títulos e resumos, seguida da leitura completa dos textos, identificamos seis estudos para inclusão.^15-17,23-25^ totalizando 233.474 participantes, dos quais 11.663 eram mulheres (Tabela 1). Os motivos para exclusão estão detalhados nos arquivos suplementares.

**Tabela 1:** – Resumo dos estudos prospectivos incluídos na metanálise

Estudo	País	Delineamento	N	Mulheres (N)	Idade, média (SD)	Tempo de seguimento (anos)	Eventos (N)	Hormônios avaliados	Desfechos
Yeap et al.^25^ 2022	Reino Unido	Coorte, prospectivo	210700	0	58,0 (IQR: 50-53)	9 anos	1061	Testosterona total e SHBG IIQ	Insuficiência Cardíaca
Njoroge et al.^17^ 2022	Estados Unidos	Coorte, prospectivo	1061	0	76,4 (5,1)	9,6 anos	368	Redução em 1 DP nos níveis de testosterona total e livre, e DHT e SHBG livre	Insuficiência Cardíaca
Zhao et al.^24^ 2018	Estados Unidos	Coorte, prospectivo	2834	2834	64,9 (8,9)	12,1 anos	103	Aumento em 1 DP nos níveis de testosterona total, livre, estradiol, S-DHEA e SHBG	Insuficiência cardíaca (ICFEP, ICFER)
Wehr et al.^23^ 2011	Alemanha	Coorte, prospectivo	2078	0	NA	7,7 anos	77	Testosteronatotal e livre, IIQ e diminuição em 1 DP nos níveis de testosterona total, livre	Insuficiência cardíaca, mortalidade
Schäfer et al.^16^ 2021	Finlândia	Coorte, prospectivo	7855	3990	48,2 (22,6) homenes e 46,9 (21,0) mulheres	13,8 anos	564	Testosterona total IIQ	Insuficiência cardíaca
Zhao et al.^15^ 2020	Estados Unidos	Coorte, prospectivo	8946	4839	62,8 (5,5) para mulheres	19,2 anos	1818	Diminuição em 1 DP nos níveis de testosterona DHEA e SHBG livre	Insuficiência cardíaca (ICFEP, ICFER)

DP: desvio padrão; IIQ: intervalo interquartil; SHBG: globulina ligadora de hormônios sexuais; DHT: di-hidrotestosterona; ICFER: insuficiência cardíaca com fração de ejeção reduzida; ICFEP: insuficiência cardíaca com fração de ejeção preservada; DHEA: desidroepiandrosterona. Todos os estudos adotaram um nível de significância estatística de 5%.

**Figura 1:**
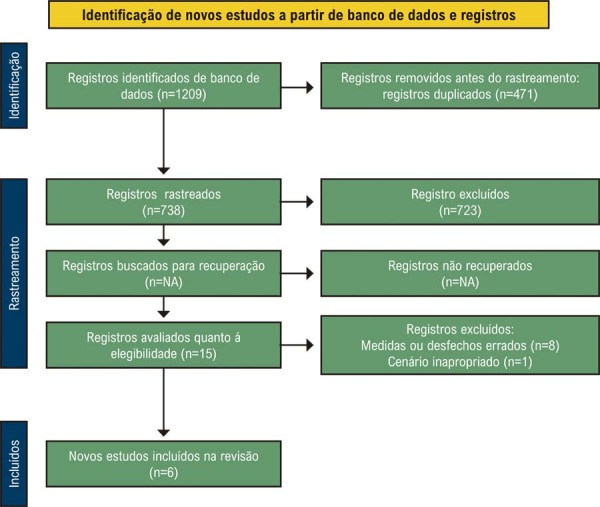
– Fluxograma PRISMA da seleção do estudo para nossa revisão sistemática. NA: Não se aplica.

Esse processo de seleção e o diagrama de fluxo PRISMA estão apresentados na Figura 1. Embora uma análise dos efeitos da DHT tenha sido pré-especificada, ela não foi realizada devido à insuficiência de dados.

Os estudos de Njoroge et al.,^17^ Wehr et al.^2 3^ e Yeap et al.^25^ focaram em populações masculinas. Njoroge et al.^17^ demonstraram que a testosterona livre estava inversamente associada à incidência de IC (HR: 1,14; IC95%: 1,01–1,28). Wehr et al.^2 3^ constataram que níveis baixos de testosterona livre estavam associados de forma independente ao aumento da mortalidade por IC. Yeap et al.^25^ relataram que concentrações mais baixas de testosterona total não estavam associadas à incidência de IC (HR: 1,15; IC95%: 0,91–1,45), mas concentrações mais baixas de SHBG estavam relacionadas a uma maior incidência de IC (HR: 0,69; IC 95%: 0,54–0,89). Zhao et al.^24^ focaram em mulheres pós-menopáusicas e constataram que a testosterona total não estava associada ao risco de eventos de IC (HR: 1,09; IC 95%: 0,90–1,34).

Schäfer et al.^16^ estudaram uma população ampla e descobriram que, após ajuste completo — incluindo índice de massa corporal e relação cintura-quadril — os níveis de testosterona não foram preditivos de IC, tanto em homens [HR: 0,99; IC 95%: 0,70–1,42; p = 0,77 para o quartil mais baixo vs. o mais alto] quanto em mulheres [HR: 0,92; IC 95%: 0,64–1,33; p = 0,99 para o quartil mais baixo vs. o mais alto]. Zhao et al.^15^ relataram HRs para IC associadas à diminuição de um desvio padrão na testosterona total log-transformada, DHEA-S e SHBG. Em homens, os HRs foram 1,10 (IC 95%: 1,03–1,17), 1,07 (IC 95%: 1,00–1,15) e 1,04 (IC 95%: 0,96–1,11), respectivamente. Em mulheres, os HRs foram 1,05 (IC 95%: 0,99–1,13), 1,17 (IC 95%: 1,09–1,24) e 0,93 (IC 95%: 0,85–1,01), respectivamente.

### Resumo da estimativa

**Figura 2:**
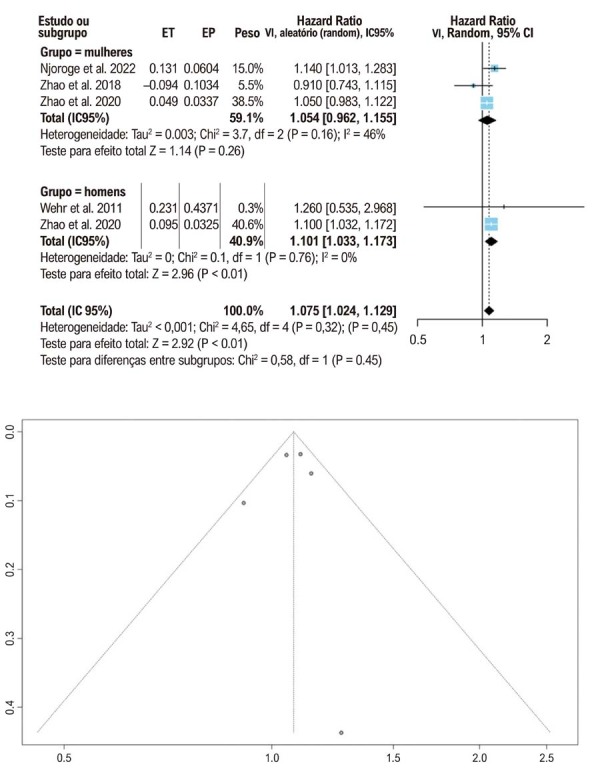
– Redução dos níveis de testosterona e insuficiência cardíaca por um desvio padrão, representada em gráficos de floresta (forest plots) e gráficos de funil (funnel plots).** ET: efeito do tratamento; EP: erro padrão; IC: intervalo de confiança; VI: variância inversa.

Testamos nossa hipótese de que os níveis hormonais basais poderiam indicar um risco aumentado de progressão para IC. Para a testosterona total, empregamos duas abordagens analíticas: análise por desvio padrão e comparação por quartis/quintis. Ao avaliar a redução dos níveis de testosterona em um desvio padrão, encontramos uma HR agrupada de 1,10 (IC 95%: 1,03–1,17), com um I^2^ de 0. Ao analisar a mesma hipótese em mulheres, o HR foi de 1,05 (IC 95%: 0,98–1,16), com um I^2^ de 46% (Figura 2).

**Figura 3:**
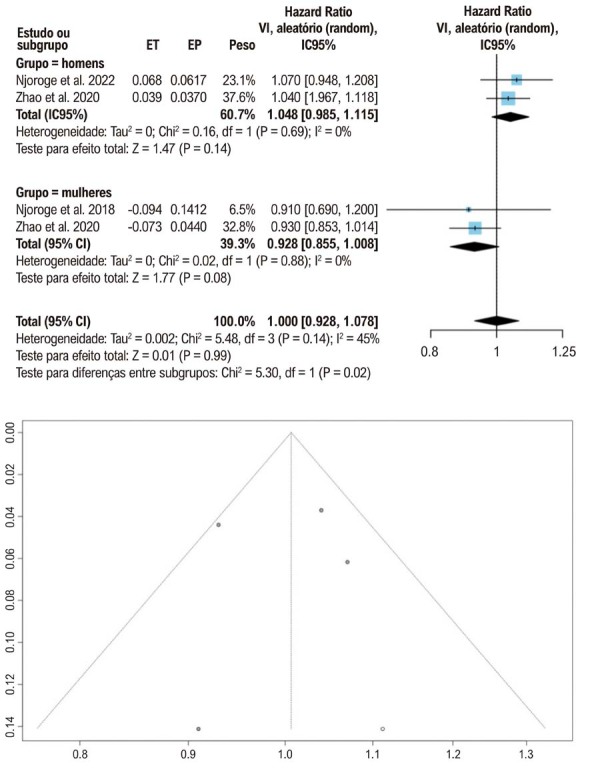
– Redução dos níveis de globulina ligadora de hormônios sexuais (SHBG) e desenvolvimento de insuficiência cardíaca por um desvio padrão, representados em gráficos de floresta (forest plots) e gráficos de funil (funnel plots).** ET: efeito do tratamento; EP: erro padrão; IC: intervalo de confiança: VI: variância inversa.

Na comparação por quartis/quintis, não foram encontradas associações estatisticamente significativas em homens ou mulheres. Isso sugere que os níveis de testosterona total, quando analisados por quartis ou quintis, não predizem significativamente o risco de IC. Em relação à SHBG, estudos que avaliaram reduções de um desvio padrão relataram HRs não significativas (Figura 3), indicando que alterações nos níveis de SHBG não são um preditor confiável do risco de IC. De modo geral, a análise sugere que, embora os níveis de testosterona total possam ter algum valor preditivo para IC em homens, o mesmo não pode ser afirmado conclusivamente para mulheres, e os níveis de SHBG não parecem ser um indicador significativo para nenhum dos sexos.

### Análise de sensibilidade

Realizamos duas análises de influência pré-especificadas do tipo LOO — uma para a meta-estimativa da testosterona total e outra para a meta-estimativa da SHBG (Figuras Suplementares S1 e S2). A omissão sequencial de cada estudo na análise da testosterona total resultou em HRs agrupadas que variaram apenas modestamente, de 1,07 a 1,11. A menor estimativa pontual foi observada após a exclusão de Njoroge et al.^17^ (2022) (HR agrupado = 1,07; IC 95%: 0,99–1,15), enquanto a maior ocorreu com a exclusão de Zhao et al.^15^ (2020, mulheres) (HR agrupado = 1,11; IC 95%: 1,03–1,20). Nenhuma coorte individual alterou substancialmente a direção ou a significância estatística da associação em homens.

A análise LOO paralela para SHBG produziu HRs agrupadas variando de 0,97 a 1,02, com todos os intervalos de confiança cruzando a unidade. A exclusão de Yeap et al.^25^ (2022) deslocou a estimativa mais afastada do valor nulo (HR = 0,97; IC 95%: 0,90–1,05), enquanto a omissão de Zhao et al.^15^ (2020, homens) aproximou ligeiramente a estimativa da unidade (HR = 1,02; IC 95%: 0,94–1,10).

### Viés de publicação

**Figura 4:**
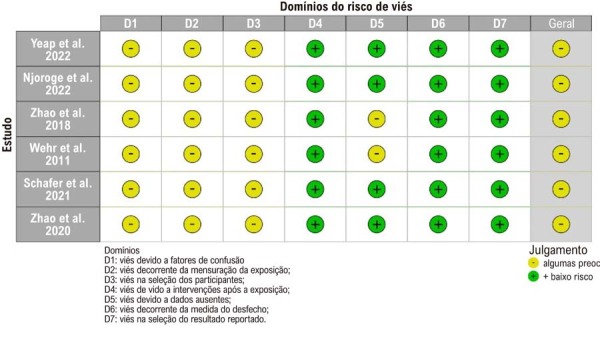
– Risco de Viés segundo a ferramenta ROBINS-E; D1: devido a fatores de confusão; D2: decorrente da mensuração da exposição; D3: seleção dos participantes; D4: devido a intervenções após a exposição; D5: devido a dados ausentes; D6: decorrente da medida do desfecho; D7: na seleção do resultado reportado. Julgamento: “–”: algumas preocupações; “+”: baixo risco de viés.

Avaliamos o risco de viés de publicação utilizando gráficos de funil, conforme ilustrado nas Figuras 2 e 3. A simetria dos gráficos sugere ausência de viés de publicação significativo. O risco de viés em cada estudo incluído foi avaliado por meio da ferramenta ROBINS-E (Figura 4).

## Discussão

Ao longo dos anos, a relação entre os níveis de testosterona e as doenças cardiovasculares tem sido controversa, com estudos apresentando resultados conflitantes. Nossa análise sugere que a testosterona pode exercer efeitos tanto benéficos quanto adversos sobre o sistema cardiovascular, os quais podem se contrabalançar, resultando em um impacto geral mínimo. Essa complexidade é ainda mais evidenciada pelo papel da testosterona como pró-hormônio que se converte em estradiol, um hormônio que também influencia a saúde cardiovascular. A ausência de uma associação clara em nosso estudo destaca a complexa interação entre os fatores hormonais na saúde cardiovascular, o que justifica estudos adicionais.^9,26^

Em indivíduos com IC, a congestão hepática pode elevar os níveis de SHBG, reduzindo assim a testosterona livre. Embora os níveis de testosterona diminuam com o aumento da gravidade da IC — sugerindo um possível papel protetor — nossos achados não sustentam um impacto clínico significativo dessas alterações hormonais. Tratamentos com testosterona exógena têm apresentado resultados variados, geralmente provenientes de estudos de curto prazo com amostras reduzidas, sem evidências claras de benefícios cardiovasculares. Isso sugere que níveis baixos de testosterona endógena podem ser mais indicativos de um estado geral de saúde comprometido do que uma causa direta da IC.^27-29^

Nossa análise revelou que uma redução nos níveis de testosterona total equivalente em um desvio padrão esteve modestamente associada a um aumento no risco de IC em homens (HR: 1,10; IC 95%: 1,03–1,17), mas não em mulheres. No entanto, ao comparar os níveis de testosterona entre quartis ou quintis, não encontramos associação significativa em nenhum dos sexos. Essa discrepância evidencia os desafios em estabelecer relações causais em estudos observacionais. Efeitos de pequena magnitude e a possibilidade de vieses mínimos anularem riscos relativos estatisticamente significativos reforçam a necessidade de uma interpretação cautelosa.

Além disso, nossa análise Bayesiana indicou evidência muito fraca para a hipótese alternativa, com um Fator de Bayes de aproximadamente 0,9885. Isso sugere que a associação observada entre baixos níveis de testosterona e aumento do risco de IC em homens não é fortemente sustentada pelos dados. No entanto, esses achados oferecem *insights* valiosos para o debate contínuo sobre o papel da testosterona na saúde cardiovascular. Embora os níveis de testosterona isoladamente possam não ser um forte preditor de risco para IC, sua interação com outros fatores fisiológicos merece mais investigações.^30^

### Limitações

Esta metanálise apresenta várias limitações. A heterogeneidade nos desenhos dos estudos e nas características das populações introduziu uma variabilidade substancial, o que pode afetar a generalização de nossos achados. Com apenas seis estudos incluídos, lidar com diferentes medidas de desfecho foi desafiador e exigiu análises adicionais não planejadas. Por exemplo, em um dos estudos (Wehr et al.^23^) incluídos em nossa metanálise, o desfecho primário foi a mortalidade relacionada à IC, e não o diagnóstico incidente de IC. Embora os pacientes desse estudo tivessem diagnóstico confirmado de IC antes do óbito, o uso da mortalidade como substituto da incidência pode ter levado à subestimação do número total de eventos de IC. Ainda assim, as análises de sensibilidade excluindo esse estudo apresentaram resultados semelhantes, reforçando a robustez de nossos achados. Apesar dessas limitações, nosso estudo ressalta a necessidade de protocolos de pesquisa mais padronizados em estudos futuros, a fim de melhorar a comparabilidade e a generalização dos resultados.

Além disso, devido ao número limitado de estudos incluídos, testes estatísticos para assimetria dos gráficos de funil — como os testes de Egger e Begg — não foram realizados, pois esses métodos são conhecidos por apresentar baixo poder estatístico quando há menos de dez estudos disponíveis, podendo gerar resultados pouco confiáveis. Por isso, optamos pela inspeção visual dos gráficos de funil, que é o método recomendado nessas condições.

Embora estudos com diagnósticos prévios de IC ou IM tenham sido excluídos, o risco de confundimento residual permanece. Variáveis não mensuradas, como predisposições genéticas, fatores de estilo de vida e tratamentos médicos concomitantes, podem influenciar tanto os níveis de testosterona quanto os desfechos relacionados à IC, potencialmente enviesando as associações observadas. Pesquisas futuras devem buscar incluir essas variáveis para isolar com maior precisão os efeitos dos níveis de testosterona sobre o risco de IC.

Por fim, o tamanho de efeito modesto observado e as evidências limitadas da análise Bayesiana sugerem que a relevância clínica da associação entre baixos níveis de testosterona e o risco de IC permanece incerta. Ainda assim, nossos achados fornecem uma base para futuras pesquisas que explorem essas associações com mais profundidade, especialmente em populações maiores e mais diversas. Ensaios clínicos randomizados bem conduzidos são particularmente necessários para confirmar essas associações e esclarecer os possíveis mecanismos subjacentes.

## Conclusão

Esta metanálise teve como objetivo quantificar o valor preditivo de baixos níveis séricos de testosterona, DHT e SHBG no desenvolvimento futuro de IC, incluindo tanto ICFER quanto ICFEP. Após um rigoroso processo de triagem, seis estudos foram incluídos. A análise revelou que uma redução de um desvio padrão nos níveis de testosterona esteve modestamente associada a um aumento no risco de IC em homens, mas não em mulheres. Os níveis de SHBG não foram preditores significativos de risco de IC (Figura Central).

**Figura Central:**
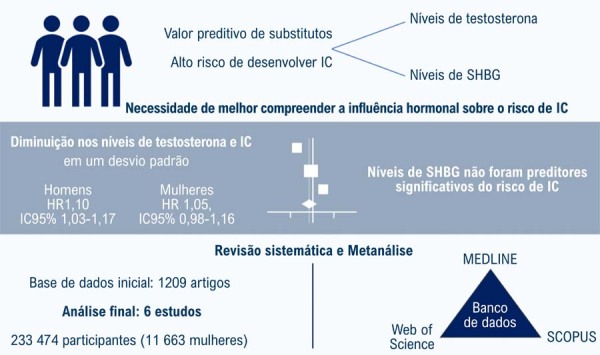
Impacto dos Baixos Níveis de Testosterona e SHBG sobre o Risco de Insuficiência Cardíaca: Uma Revisão Sistemática e Metanálise

Impacto de baixos níveis de testosterona e globulina ligadora de hormônios sexuais (SHBG) no risco de insuficiência cardíaca. Níveis baixos de testosterona estão modestamente associados ao aumento do risco de insuficiência cardíaca em homens, enquanto os níveis de SHBG não foram preditores significativos de risco de insuficiência cardíaca. IC: intervalo de confiança; HR: razão de risco.

Embora nossos achados indiquem uma possível associação entre baixos níveis de testosterona e aumento do risco de IC em homens, as evidências permanecem fracas, conforme demonstrado pela análise Bayesiana. A heterogeneidade entre os estudos e os pequenos tamanhos de efeito dificultam ainda mais a interpretação. Esses resultados reforçam a necessidade de pesquisas mais robustas para esclarecer o papel da testosterona baixa e da SHBG no desenvolvimento da IC. Ensaios clínicos randomizados bem desenhados são especialmente necessários para confirmar essas associações e elucidar os mecanismos subjacentes.

## Material suplementar

Folha 1 - supplementarytable_ex

Figure S1

Figure S2

## References

[1] GBD 2016 Disease and Injury Incidence and Prevalence Collaborators (2017). Global, Regional, and National Incidence, Prevalence, and Years Lived with Disability for 328 Diseases and Injuries for 195 Countries, 1990-2016: A Systematic Analysis for the Global Burden of Disease Study 2016. Lancet.

[2] Maggioni AP, Orso F, Calabria S, Rossi E, Cinconze E, Baldasseroni S (2016). The Real-World Evidence of Heart Failure: Findings from 41 413 Patients of the ARNO Database. Eur J Heart Fail.

[3] Benjamin EJ, Muntner P, Alonso A, Bittencourt MS, Callaway CW, Carson AP (2019). Heart Disease and Stroke Statistics-2019 Update: A Report from the American Heart Association. Circulation.

[4] Chun S, Tu JV, Wijeysundera HC, Austin PC, Wang X, Levy D (2012). Lifetime Analysis of Hospitalizations and Survival of Patients Newly Admitted with Heart Failure. Circ Heart Fail.

[5] Hartupee J, Mann DL (2017). Neurohormonal Activation in Heart Failure with Reduced Ejection Fraction. Nat Rev Cardiol.

[6] Zipes DP, Libby P, Mann DL, Tomaselli GF, Bonow RO (2018). Braunwald's Heart Disease: A Textbook of Cardiovascular Medicine.

[7] McDonagh TA, Metra M, Adamo M, Gardner RS, Baumbach A, Böhm M (2021). 2021 ESC Guidelines for the Diagnosis and Treatment of Acute and Chronic Heart Failure. Eur Heart J.

[8] Araujo AB, Dixon JM, Suarez EA, Murad MH, Guey LT, Wittert GA (2011). Clinical Review: Endogenous Testosterone and Mortality in Men: A Systematic Review and Meta-Analysis. J Clin Endocrinol Metab.

[9] Ruige JB, Mahmoud AM, De Bacquer D, Kaufman JM (2011). Endogenous Testosterone and Cardiovascular Disease in Healthy Men: A Meta-Analysis. Heart.

[10] Buergel T, Steinfeldt J, Ruyoga G, Pietzner M, Bizzarri D, Vojinovic D (2022). Metabolomic Profiles Predict Individual Multidisease Outcomes. Nat Med.

[11] Saccà L (2009). Heart Failure as a Multiple Hormonal Deficiency Syndrome. Circ Heart Fail.

[12] Cittadini A, Salzano A, Iacoviello M, Triggiani V, Rengo G, Cacciatore F (2021). Multiple Hormonal and Metabolic Deficiency Syndrome Predicts Outcome in Heart Failure: The T.O.S.CA. Registry. Eur J Prev Cardiol.

[13] Subramanya V, Zhao D, Ouyang P, Lima JA, Vaidya D, Ndumele CE (2018). Sex Hormone Levels and Change in Left Ventricular Structure Among Men and Post-Menopausal Women: The Multi-Ethnic Study of Atherosclerosis (MESA). Maturitas.

[14] Luo S, Au Yeung SL, Zhao JV, Burgess S, Schooling CM (2019). Association of Genetically Predicted Testosterone with Thromboembolism, Heart Failure, and Myocardial Infarction: Mendelian Randomisation Study in UK Biobank. BMJ.

[15] Zhao D, Guallar E, Ballantyne CM, Post WS, Ouyang P, Vaidya D (2020). Sex Hormones and Incident Heart Failure in Men and Postmenopausal Women: The Atherosclerosis Risk in Communities Study. J Clin Endocrinol Metab.

[16] Schäfer S, Aydin MA, Appelbaum S, Kuulasmaa K, Palosaari T, Ojeda F (2021). Low Testosterone Concentrations and Prediction of Future Heart Failure in Men and in Women: Evidence from the Large FINRISK97 Study. ESC Heart Fail.

[17] Njoroge JN, Tressel W, Biggs ML, Matsumoto AM, Smith NL, Rosenberg E (2022). Circulating Androgen Concentrations and Risk of Incident Heart Failure in Older Men: The Cardiovascular Health Study. J Am Heart Assoc.

[18] Page MJ, McKenzie JE, Bossuyt PM, Boutron I, Hoffmann TC, Mulrow CD (2021). The PRISMA 2020 Statement: An Updated Guideline for Reporting Systematic Reviews. BMJ.

[19] Steel P, Fariborzi H, Hendijani R (2023). An Application of Modern Literature Review Methodology: Finding Needles in Ever-Growing Haystacks.

[20] Microsoft Corporation (2024). Microsoft Excel.

[21] Higgins JPT, Morgan RL, Rooney AA, Taylor KW, Thayer KA, Silva RA (2024). A Tool to Assess Risk of Bias in Non-Randomized Follow-Up Studies of Exposure Effects (ROBINS-E). Environ Int.

[22] Viechtbauer W (2010). Conducting Meta-Analyses in R with the Metafor Package. J Stat Softw.

[23] Wehr E, Pilz S, Boehm BO, März W, Grammer T, Obermayer-Pietsch B (2011). Low Free Testosterone is Associated with Heart Failure Mortality in Older Men Referred for Coronary Angiography. Eur J Heart Fail.

[24] Zhao D, Guallar E, Ouyang P, Subramanya V, Vaidya D, Ndumele CE (2018). Endogenous Sex Hormones and Incident Cardiovascular Disease in Post-Menopausal Women. J Am Coll Cardiol.

[25] Yeap BB, Marriott RJ, Antonio L, Raj S, Dwivedi G, Reid CM (2022). Associations of Serum Testosterone and Sex Hormone-Binding Globulin with Incident Cardiovascular Events in Middle-Aged to Older Men. Ann Intern Med.

[26] Tivesten A, Bollano E, Nyström HC, Alexanderson C, Bergström G, Holmäng A (2006). Cardiac Concentric Remodelling Induced by Non-Aromatizable (Dihydro-)Testosterone is Antagonized by Oestradiol in Ovariectomized Rats. J Endocrinol.

[27] Kloner RA, Carson C, Dobs A, Kopecky S, Mohler ER (2016). Testosterone and Cardiovascular Disease. J Am Coll Cardiol.

[28] Iellamo F, Volterrani M, Caminiti G, Karam R, Massaro R, Fini M (2010). Testosterone Therapy in Women with Chronic Heart Failure: A Pilot Double-Blind, Randomized, Placebo-Controlled Study. J Am Coll Cardiol.

[29] Elagizi A, Köhler TS, Lavie CJ (2018). Testosterone and Cardiovascular Health. Mayo Clin Proc.

[30] Grimes DA, Schulz KF (2012). False Alarms and Pseudo-Epidemics: The Limitations of Observational Epidemiology. Obstet Gynecol.

