# Supplementary material for: Impacto dos Baixos Níveis de Testosterona e SHBG sobre o Risco de Insuficiência Cardíaca: Uma Revisão Sistemática e Metanálise
Source: Arq Bras Cardiol. 2025 Oct 29;122(10):e20250244. [Article in Portuguese] doi: 10.36660/abc.20250244 (PMC12677805; doi:10.36660/abc.20250244)

Figure S1. Leave-one-out sensitivity analysis of the decrement of testosterone levels and HF by one standard deviation.

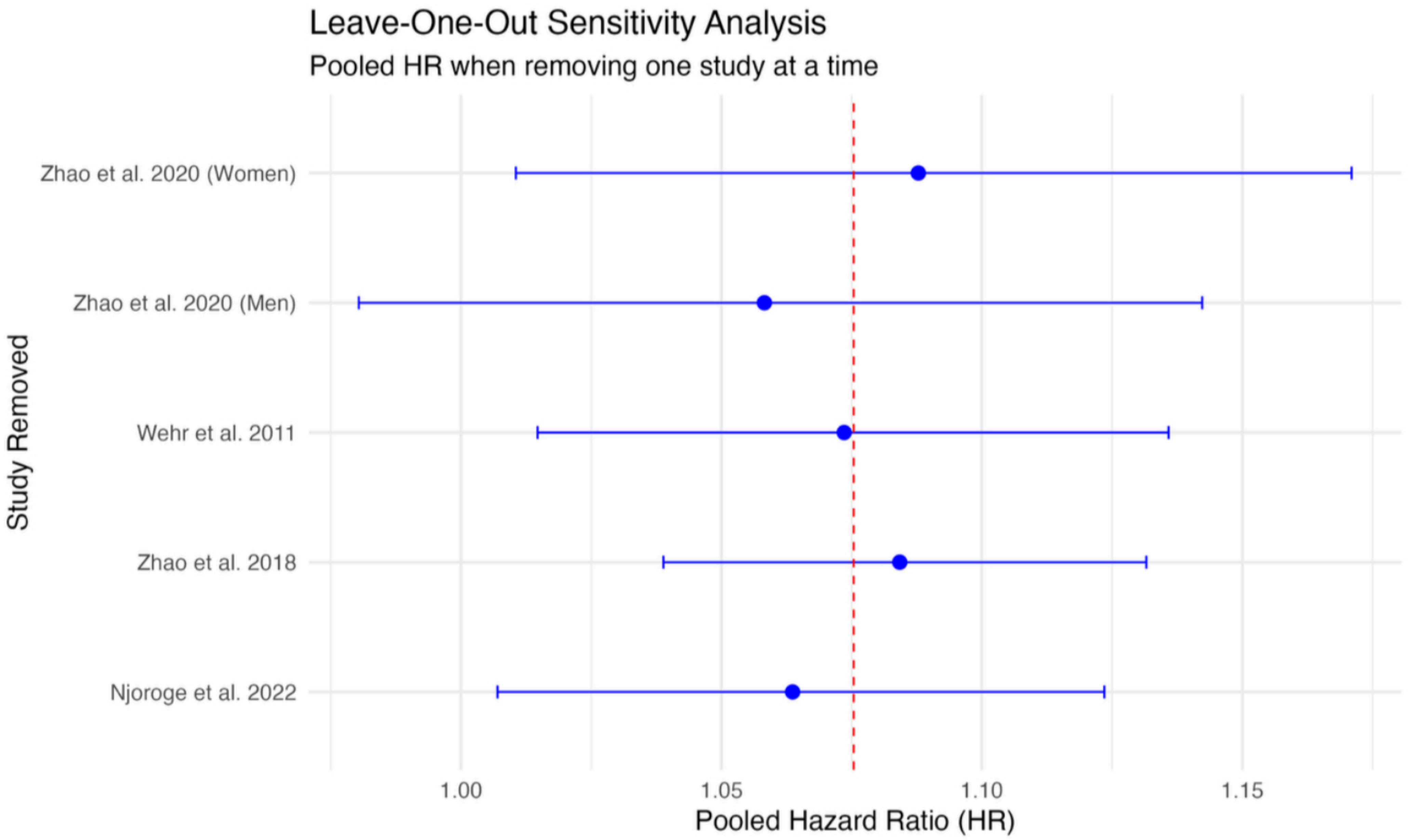

Supplement: Figure S1 [file 0066-782x-abc-122-10-e20250244-suppl02.pdf]
