# Supplementary material for: Impacto dos Baixos Níveis de Testosterona e SHBG sobre o Risco de Insuficiência Cardíaca: Uma Revisão Sistemática e Metanálise
Source: Arq Bras Cardiol. 2025 Oct 29;122(10):e20250244. [Article in Portuguese] doi: 10.36660/abc.20250244 (PMC12677805; doi:10.36660/abc.20250244)
Supplement: Translation

# Translation: Impact of Low Testosterone and SHBG Levels on Heart Failure Risk: A Systematic Review and Meta-Analysis

**DOI:** 10.36660/abc.20250244i

**Authors:** Thiago Artioli, Layane Bonfante Batista, Kleber Franchini, José Nunes de Alencar

**Affiliations:** 1 Brazil Instituto Dante Pazzanese de Cardiologia, São Paulo, SP – Brazil

**Keywords:** Heart Failure, Testosterone, Biomarkers, Risk Factors

## Abstract

**Background:**

Studies suggest a possible link between low levels of testosterone and sex hormone binding globulin (SHBG) and adverse cardiovascular outcomes; however, this relationship remains poorly defined.

**Objectives:**

This systematic review aimed to evaluate the predictive value of baseline levels of testosterone, dihydrotestosterone (DHT), and SHBG for the incidence of heart failure (HF), providing deeper insight into the hormonal influence on HF risk.

**Methods:**

We conducted a comprehensive search of the MEDLINE, Scopus, and Web of Science databases to identify cohort and nested case-control studies that measured hormone levels in adults without prior HF. Risk of bias was assessed using the ROBINS-E tool. Pooled hazard ratios (HRs) and odds ratios (ORs) were estimated using bivariate random-effects models. A statistical significance level of 0.05 was applied to all analyses.

**Results:**

Out of 1,209 articles screened, 738 remained after deduplication. Six studies, including 233,474 participants (11,663 women), met the inclusion criteria. A one standard deviation decrease in testosterone levels was modestly associated with an increased risk of HF in men (HR 1.10, 95% CI: 1.03-1.17), but not in women (HR 1.05, 95% CI: 0.98-1.16). Comparisons across quartiles or quintiles did not reveal significant associations, and SHBG levels were not significant predictors of HF risk. Bayesian analysis provided weak evidence for the association (Bayes factor = 0.99).

**Conclusions:**

This meta-analysis suggests that low testosterone levels are modestly associated with an increased risk of HF in men, highlighting a potentially important yet underexplored aspect of cardiovascular health. The heterogeneity in study designs and population characteristics, combined with the weak associations observed, underscores the need for further rigorous investigation. Well-designed randomized controlled trials are essential to confirm these findings and to elucidate the underlying biological mechanisms.

## Introduction

Heart failure (HF) remains a major global health issue, affecting over 60 million people worldwide.^1,2^ Despite advances in treating HF with reduced ejection fraction (HFrEF), patients continue to face significant risks of disease progression and adverse outcomes, even with guideline-directed medical therapy.^3,4^

Neurohormonal mechanisms, particularly the activation of the sympathetic nervous system and the renin-angiotensin system, are central to HF progression and closely linked to morbidity and mortality.^5^ Therapies targeting these systems have improved HF management. However, there is growing evidence that downregulation of various hormones and metabolic signals also contributes to HF progression.

Approximately 25% of men with chronic HF exhibit testosterone deficiency, along with reduced levels of the adrenal androgen dehydroepiandrosterone (DHEA) and its sulfate. These deficiencies correlate with HF severity and are linked to symptoms such as reduced muscle mass, cachexia, depression, and fatigue.^6-9^

Recent studies have investigated the relationship between serum testosterone, dihydrotestosterone (DHT), sex hormone-binding globulin (SHBG) levels, and HF, encompassing both HFrEF and HF with preserved ejection fraction (HFpEF).^10-13^ However, these findings are often contradictory and lack comprehensive analysis, leaving gaps in our understanding of the specific impacts of hormonal deficiencies on HF.^14-17^

As the population ages and hypogonadism becomes more prevalent, elucidating the role of testosterone and related hormones in HF is of growing importance. This systematic review and meta-analysis seeks to quantitatively evaluate the predictive value of low serum levels of testosterone, DHT, and/or SHBG for future HF risk, providing clearer insights into their potential as biomarkers for HF progression and informing future research directions.

## Methods

This systematic review protocol is registered with the International Prospective Register of Systematic Reviews (PROSPERO): registration number CRD42024550371. The study received no external funding, and the authors declare no competing interests. All data used in this review are available from the authors.

### Study eligibility criteria

We included cohort or nested case-control studies that measured serum levels of testosterone, DHT and/or SHBG in a general population with no history of HF. Case reports, reviews, and meta-analysis were excluded. At the end of the follow-up period, individuals with low serum levels of testosterone, DHT and/or SHBG were compared to those with normal levels regarding the incidence of HF, including HFrEF and HFpEF. The year of publication and specific follow-up duration were not considered exclusion criteria. Studies that did not measure or reported the outcomes of interest were deemed ineligible.

### Participants

The study population consisted of adults with baseline serum levels of testosterone, DHT and/or SHGB, who were subsequently followed for the development of newly diagnosed HF, including HFrEF or HFpEF. The hormones were measured using immunoassay or mass spectrometry. We excluded patients with a previous diagnosis of HF or myocardial infarction, pregnant women, patients with a current or past diagnosis of cancer (e.g., prostate cancer), and those undergoing testosterone replacement therapy.

### Information source and search strategy

We conducted a comprehensive search of MEDLINE, Scopus and Web of Science. No restrictions were applied regarding publication year or language, ensuring an inclusive scope. Keywords and MeSH terms used in the search included testosterone, DHT, SHBG, HF, HFrEF and HFpEF. This review follows the Preferred Reporting Items for Systematic Reviews and Meta-Analyses (PRISMA) Guidelines.^18^

### Selection and data collection

Titles and abstracts were independently screened by two reviewers (T.A. and L.B.B.) to identify eligible studies, with any disagreements resolved by a third reviewer. Full-text screening was also conducted independently by two researchers (J.A. and A.S.). Data extraction, facilitated by HubMeta software,^19^ included study characteristics such as authorship, publication year, country of origin and study design. Key variables extracted included serum levels of testosterone, DHT and/or SHBG; the assays used; study design; outcomes assessed; confounders adjusted for mean age of the study population; follow-up duration; country of study and year of publication. Studies were required to provide sufficient information to estimate a hazard ratio (HR) with a 95% confidence interval (CI). We extracted the HR estimates that were adjusted for the highest number of potential confounding variables. Searches and analyses were performed independently by the investigators. Data tabulation was conducted using Excel Software.^20^

### Risk of bias

The risk of bias was assessed using the Risk Of Bias In Non-randomized Studies - of Exposures (ROBINS-E) tool.^21^

### Sensitivity analyses

In prespecified sensitivity analyses, we analyzed data related to various laboratory measures of testosterone, DHT, and SHBG levels. In addition, to evaluate the influence of each individual study on the pooled estimates, we performed a leave-one-out (LOO) influence analysis, by sequentially removing one study at a time and recalculating the summary HR. This approach was used to evaluate the robustness of our findings in light of variability in laboratory measurements.

### Data synthesis and effect measures

In our meta-analysis, we employed a bivariate random-effects model to pool odds ratio estimates across studies. This model was selected for its ability to account for both within-study and between-study variability. The analyses were conducted using the R / OnlineMeta software.^22^ Forest plots were generated to visually display distribution of the odds ratio across studies and their pooled estimates, providing a clear graphical summary of the meta-analysis results. A statistical significance level of 0.05 was applied to all analyses.

## Results

### Eligible Studies

Our initial database search yielded 1,209 articles. After deduplication, 738 articles remained. Following title and abstract screening, and full-text review, we identified six studies for inclusion,^15-17,23-25^ comprising a total of 233,474 participants, of whom 11,663 were women (Table 1). Reasons for exclusion are detailed in the supplementary files.

**Table 1:** – Summary of prospective studies included in meta-analysis

Study	Country	Study Design	N	Female (N)	Age, mean (SD)	Follow-up Duration (Years)	Events (N)	Hormones Evaluated	Outcomes
Yeap et al. ^25^ 2022	United Kingdom	Prospective cohort	210700	0	58.0 (IQR: 50-53)	9.0 years	1061	Total testosterone and SHBG IQR	Heart failure
Njoroge et al. ^17^ 2022	United States of America	Prospective cohort	1061	0	76.4 (5.1)	9.6 years	368	Decrease in 1SD of total and Free testosterone, total and free DHT, SHBG	Heart failure
Zhao et al.^24^ 2018	United States of America	Prospective cohort	2834	2834	64.9 (8.9)	12.1 years	103	Increase in 1 SD of total and Free testosterone, Estradiol, S-DHEA and SHBG	Heart failure (HFpEF, HFrEF)
Wehr et al.^23^ 2011	Germany	Prospective cohort	2078	0	NA	7.7 years	77	Total and free testosterone IQR and decrease in 1 SD of total testosterone and free testosterone	Heart failure mortality
Schafer et al.^16^ 2021	Finland	Prospective cohort	7855	3990	48.2 (22.6) men and 46.9 (21.0) women	13.8 years	564	Total testosterone IQR	Heart failure
Zhao et al.^15^ 2020	United States of America	Prospective cohort	8946	4839	62.8 (5.5) for women	19.2 years	1818	Decrease in 1SD of Total Testosterone, DHEA and SHBG	Heart failure (HFpEF, HFrEF)

SD: standard deviation; IQR: interquartile range; SHBG: sex hormone-binding globulin; DHT: dihydrotestosterone; HFrEF: heart failure with reduced ejection fraction; HFpEF: heart failure with preserved ejection fraction; DHEA: dehydroepiandrosterone. All studies adopted 5% statistical significance.

This selection process and the PRISMA flow diagram are presented in Figure 1. Although an analysis of DHT effects was pre-specified, it was not performed due to insufficient data.

**Figure 1:** – PRISMA Flowchart of study selection for our systematic review. NA: Not applicable.

Studies by Njoroge et al.,^17^ Wehr et al.^23^ and Yeap et al.^25^ focused on male population. Njoroge et al.^17^ demonstrated that free testosterone was inversely associated with incident HF (HR 1.14; 95% CI 1.01–1.28). Wehr et al.^23^ found that low free testosterone levels were independently associated with increased HF mortality. Yeap et al.^25^ reported that lower total testosterone concentrations were not associated with incident HF (HR, 1.15; 95%CI 0.91 – 1.45), but lower SHBG concentrations were linked to a higher incidence of HF (HR, 0.69; 95%CI 0.54 – 0.89). Zhao et al.^24^focused on post-menopausal women and found that total testosterone was not associated with risk for HF events (HR 1.09 [95% CI: 0.90 to 1.34]).

Schäfer et al.^16^ studied a broad population and found that, after full adjustment – including for body mass index and waist-to-hip ratio – testosterone levels were not predictive of incident HF, neither in men [HR: 0.99; 95% CI: 0.70–1.42; p = 0.77 for lowest vs. highest quartile] nor in women [HR: 0.92; 95% CI: 0.64–1.33; p = 0.99 for lowest vs. highest quartile]. Zhao et al.^15^ reported HRs for HF associated with a one standard deviation (SD) decrease in log-transformed total testosterone, DHEA-S, and SHBG. In men, the HRs were 1.10 (95% CI: 1.03–1.17), 1.07 (95% CI: 1.00–1.15), and 1.04 (95% CI: 0.96–1.11), respectively. In women, the HRs were 1.05 (95% CI: 0.99–1.13), 1.17 (95% CI: 1.09–1.24), and 0.93 (95% CI: 0.85–1.01), respectively.

### Summary estimate

We tested our hypothesis that baseline hormone levels could indicate a higher risk of progressing to HF. For total testosterone, we employed two analytical approaches: standard deviation analysis and quartile/quintile comparison. Evaluating the decrement of testosterone levels by one standard deviation, we found a pooled HR of 1.10 (95% CI: 1.03-1.17), with an I^2^ of 0. When analyzing the same hypothesis in women, the HR was 1.05 (95% CI: 0.98-1.16), with an I^2^ of 46% (Figure 2).

**Figure 2:** – Decrement of testosterone levels and heart failure by one standard deviation in forest plots and funnel plots graphics. TE: treatment effect; SE: standard error; CI: confidence interval; IV: inverse variance.

In the quartile/quintile comparison, no statistically significant associations were found in either men or women. This suggests that total testosterone levels, when analyzed across quartiles or quintiles, do not significantly predict the risk of HF. Regarding SHBG, studies evaluating decreases of one standard deviation reported non-significant hazard ratios (HRs) (Figure 3), indicating that changes in SHBG levels are not a reliable predictor of HF risk. Overall, the analysis suggests that while total testosterone levels may have some predictive value for HF in men, the same cannot be conclusively stated for women, and SHBG levels do not appear to be a significant indicator for either sex.

**Figure 3:** – Decrement of sex hormone-binding globulin levels and HF development by one standard deviation in forest plots and funnel plots graphics. TE: treatment effect; SE: standard error; CI: confidence interval; IV: inverse variance.

### Sensitivity analysis

We performed two prespecified LOO influence analyses - one for the total testosterone meta-estimate and another for the SHBG meta-estimate (Supplementary Figures S1 and S2). Sequential omission of each study from the total testosterone analysis yielded pooled HRs that varied only modestly, ranging from 1.07 to 1.11. The lowest point estimate was observed after excluding Njoroge et al.^17^ (2022), (pooled HR = 1.07, 95 % CI 0.99–1.15), whereas the highest occurred when Zhao et al.^15^ (2020, women) was excluded (pooled HR = 1.11, 95 % CI 1.03–1.20). No single cohort substantially altered the direction or statistical significance of the association in men.

The parallel LOO analysis for SHBG produced pooled HRs ranging from 0.97 to 1.02, with all confidence intervals crossing unity. Excluding Yeap et al.^25^ (2022) shifted the estimate furthest from the null (HR = 0.97, 95 % CI 0.90–1.05), whereas omission of Zhao et al.^15^ (2020, men) moved the estimate slightly toward unity (HR = 1.02, 95 % CI 0.94–1.10).

### Publication bias

We assessed the risk of publication bias using funnel plots, as illustrated in Figures 2 and 3. The symmetry of the plots suggests no significant publication bias. We evaluated the risk of bias in each included study using the ROBINS-E tool (Figure 4).

**Figure 4:** – ROBINS-E Risk of Bias. D1: due to confounding; D2: arising from measurement of the exposure; D3: selection of participants; D4: due to post-exposure interventions; D5: due to missing data; D6: arising from measurement of the outcome; D7: in selection of the reported result. Judgement: “-“ some concerns; and “+” low risk of bias.

## Discussion

Over the years, the relationship between testosterone levels and cardiovascular diseases has been contentious, with studies reporting conflicting results. Our analysis suggests that testosterone may exert both beneficial and adverse effects on the cardiovascular system, which could offset each other, resulting in a minimal overall impact This complexity is further highlighted by testosterone’s role as a pro-hormone that converts to estradiol, a hormone that also influences cardiovascular health. The lack of a clear-cut association in our study underscores the intricate interplay of hormonal factors in cardiovascular health, warranting further investigation.^9,26^

In individuals with HF, liver congestion may elevate SHBG levels, thereby reducing free testosterone. Although testosterone levels decrease with increasing HF severity, suggesting a potential protective role, our findings do not support a significant clinical impact of these hormonal changes. Exogenous testosterone treatments have yielded mixed results, often derived from short-term studies with small sample sizes, and have failed to demonstrate clear cardiovascular benefits. This suggests that low endogenous testosterone levels may serve more as a marker of overall poor health rather than a direct cause of HF.^27-29^

Our analysis revealed that a decrease in total testosterone levels by one standard deviation was modestly associated with an increased risk of HF in men (HR: 1.10, 95% CI: 1.03-1.17) but not in women. However, when comparing testosterone levels across quartiles or quintiles, we found no significant association in either sex. This discrepancy highlights the challenges in establishing causal relationships in observational studies. Small effect sizes and the potential for minimal biases to negate statistically significant relative risks emphasize the need for cautious interpretation.

Furthermore, our Bayesian analysis indicated very weak evidence for the alternative hypothesis, with a Bayes Factor of approximately 0.9885. This suggests that the observed association between low testosterone levels and increased HF risk in men is not strongly supported by the data**.** However, these findings offer valuable insights into the ongoing debate about the role of testosterone in cardiovascular health. While testosterone levels alone may not serve as a strong predictor of HF risk, their interaction with other physiological factors merits further investigation.^30^

### Limitations

This meta-analysis has several limitations. Heterogeneity in study designs and population characteristics introduced considerable variability, which may affect the generalizability of our findings. With only six studies included, managing different outcomes posed challenges and required unplanned additional analyses. For example, in one of the studies (Wehr et al.^23^) included in our metanalysis, the primary outcome was HF-related mortality, rather than incident HF diagnosis. Although patients in that study had confirmed HF prior to death, the use of mortality as a proxy for incidence may have led to an underestimation of the total number of HF events. However, sensitivity analyses excluding this study yielded similar results, supporting the robustness of our findings. Despite these limitations, our study highlights the need for more standardized research protocols in future studies to enhance comparability and generalizability.

Additionally, due to the limited number of included studies, statistical tests for funnel plot asymmetry – such as Egger’s and Begg’s tests – were not performed, as these methods are known to have low statistical power when fewer than ten studies are available and may produce unreliable results. Consequently, we relied on visual inspection of funnel plots, which is the recommended approach under such conditions.

Although studies with prior diagnoses of HF or myocardial infarction were excluded, residual confounding remains a concern. Unmeasured variables – Including genetic predispositions, lifestyle factors, and concurrent medical treatments – may influence both testosterone levels and HF outcomes, potentially biasing the observed associations. Future research should aim to account for these variables to more accurately isolate the effect of testosterone levels on HF risk.

Lastly, the modest effect size observed and the limited evidence from the Bayesian analysis suggest that the clinical relevance of the association between low testosterone levels and HF risk remains uncertain. Nevertheless, our findings provide a basis for future research to investigate these associations more thoroughly, particularly in larger and more diverse populations. Well-designed randomized controlled trials are especially needed to validate these associations and uncover potential underlying mechanisms.

## Conclusion

This meta-analysis aimed to quantify the predictive value of low serum levels of testosterone, DHT, and SHBG on future HF, including both HFrEF and HFpEF. Following a rigorous screening process, six studies were included. The analysis revealed that a one standard deviation decrease in testosterone levels was modestly associated with an increased risk of HF in men, but not in women. SHBG levels were not significant predictors of HF risk (see Central Illustration).

**Central Illustration:** Impact of Low Testosterone and SHBG Levels on Heart Failure Risk: A Systematic Review and Meta-Analysis Impact of Low Testosterone and SHBG Levels on Heart Failure Risk. Low testosterone levels are modestly associated with increased risk of heart failure in men, while sex hormone-binding globulin levels were not significant predictors of heart failure risk. CI: confidence interval; HR: hazard ratio; SHBG: sex hormone-binding globulin.

Although our findings indicate some association between low testosterone levels and increased HF risk in men, the evidence remains weak, as shown by our Bayesian analysis. Study heterogeneity and small effect sizes further complicate interpretation. These results highlight the need for more robust research to fully understand the role of low testosterone and SHBG in HF development. Well-conducted randomized controlled trials are particularly necessary to confirm these associations and elucidate the underlying mechanisms.

## Supplemental Materials

Folha 1 - supplementarytable_ex

Figure S1

Figure S2
